# Comparative Genomics Search for Losses of Long-Established Genes on the Human Lineage

**DOI:** 10.1371/journal.pcbi.0030247

**Published:** 2007-12-14

**Authors:** Jingchun Zhu, J. Zachary Sanborn, Mark Diekhans, Craig B Lowe, Tom H Pringle, David Haussler

**Affiliations:** 1 Center for Biomolecular Science and Engineering, University of California Santa Cruz, Santa Cruz, California, United States of America; 2 Howard Hughes Medical Institute, University of California Santa Cruz, Santa Cruz, California, United States of America; Washington University, United States of America

## Abstract

Taking advantage of the complete genome sequences of several mammals, we developed a novel method to detect losses of well-established genes in the human genome through syntenic mapping of gene structures between the human, mouse, and dog genomes. Unlike most previous genomic methods for pseudogene identification, this analysis is able to differentiate losses of well-established genes from pseudogenes formed shortly after segmental duplication or generated via retrotransposition. Therefore, it enables us to find genes that were inactivated long after their birth, which were likely to have evolved nonredundant biological functions before being inactivated. The method was used to look for gene losses along the human lineage during the approximately 75 million years (My) since the common ancestor of primates and rodents (the euarchontoglire crown group). We identified 26 losses of well-established genes in the human genome that were all lost at least 50 My after their birth. Many of them were previously characterized pseudogenes in the human genome, such as *GULO* and *UOX*. Our methodology is highly effective at identifying losses of single-copy genes of ancient origin, allowing us to find a few well-known pseudogenes in the human genome missed by previous high-throughput genome-wide studies. In addition to confirming previously known gene losses, we identified 16 previously uncharacterized human pseudogenes that are definitive losses of long-established genes. Among them is *ACYL3*, an ancient enzyme present in archaea, bacteria, and eukaryotes, but lost approximately 6 to 8 Mya in the ancestor of humans and chimps. Although losses of well-established genes do not equate to adaptive gene losses, they are a useful proxy to use when searching for such genetic changes. This is especially true for adaptive losses that occurred more than 250,000 years ago, since any genetic evidence of the selective sweep indicative of such an event has been erased.

## Author Summary

One of the most important questions in biology is to identify the genetic changes underlying evolution, especially those along the lineage leading to the modern human. Although counterintuitive, losing a gene might actually bring a selective advantage to the organism. This type of gene loss is called adaptive gene loss. Although a few cases have been characterized in the literature, this is the first study to address adaptive gene losses on a scale of the whole human genome and a time period of up to 75 million years. The difficulty of identifying adaptive gene losses is in part the large number of pseudogenes in the human genome. To circumvent this problem, we used two methods to enrich the process for the adaptive candidates. The first is a novel approach for pseudogene detection that is highly sensitive in identifying single-copy pseudogenes that bear no apparent sequence homology to any functional human genes. Second, we used the length of time a gene is functional before loss as a proxy for biological importance, which allows us to differentiate losses of long-established genes from mere losses due to functional redundancy after gene duplication.

## Introduction

It is intuitive to think that changes leading to increased complexity, adaptation, and intelligence are achieved by the gain and improvement of genetic components such as genes and regulatory elements. However, in certain scenarios, a loss of function can also bring a selective advantage. The best-known examples are losses of cell surface receptors to confer pathogenic resistance, such as the inactivation of the *DUFFY* gene contributing to malaria resistance [1] and homozygosity for a null allele of chemokine receptor *CCR5* conveying resistance to infection by various pathogens, including HIV [2]. In addition, the loss of an existing biological component can open new developmental opportunities. The human-specific loss of a myosin heavy chain isoform expressed in the masticatory muscles has been linked to the weakening of human jaw muscles, possibly allowing the increase of cranial capacity in humans, although this is still quite speculative [3]. Adaptive gene loss is the type of genetic change that leads to better fitness for an organism by inactivating a functional gene. As argued by the “less-is-more” hypothesis, gene losses may be an important engine of evolutionary innovation [4].

In addition to adaptive evolution, gene losses can play an important role in human diseases where conditionally advantageous mutations improve fitness in a particular environment. For example, deleterious mutations affecting hemoglobin and other red blood cell proteins are common in many human populations due to a heterozygote advantage in malaria epidemic environments. This improved fitness comes at a cost for those born with deleterious mutations on both alleles, since the homozygous state causes anemia including sickle cell disease [5–7]. Other human diseases such as glucose-6-phosphate dehydrogenase deficiency [7] and cystic fibrosis [8,9] have also been associated with the heterozygote advantage.

Despite the apparent importance of adaptive gene loss, we know surprisingly little about its contribution and significance at the genomic level and over a broad time scale. Most research on adaptive evolution in mammals focuses on new genes or regulatory elements as well as on modifications to known genes, such as amino acid substitutions [10,11]. With the complete genomes of human and several other mammals including chimp, rhesus, mouse, rat, and dog [12–16], it is now feasible to systematically identify adaptive gene losses in the human lineage through the course of mammalian evolution.

A claim for adaptive genetic change typically requires evidence of DNA signatures indicating directional selection, and is accompanied by the identification of selective pressures acting on the organisms that are consistent with DNA, fossil, or historical evidence. Methods for detecting amino acid or DNA signatures left by natural selection are not generally applicable for identifying adaptive gene loss [17,18]. An inactivated gene is no longer maintained through the forces of natural selection, and secondary mutations begin to accumulate at the neutral rate. Therefore, methods based on sequence conservation or ratio of synonymous versus nonsynonymous mutations are not suitable to detect adaptive gene losses [11,19–21]. Recent adaptive losses can be detected by the distinct DNA signatures left by positive selection; however, those signatures only persist for a narrow evolutionary window of at most 250,000 years [22–24]. To detect adaptive gene losses further back into the evolutionary past, it is reasonable to assume that a nonredundant gene that was functional for a long time and then inactivated is a good candidate for adaptive gene loss. While not every loss of a well-established gene is adaptive, searching for those candidates can be used to enrich for adaptive gene losses.

Gene loss normally leaves behind a pseudogene. However, the vast majority of pseudogenes in a genome did not bring a selective advantage to the organism. Most pseudogenes arise through a gene copying operation of either retrotransposition (reverse-transcribing a processed mRNA back to DNA, which is reinserted in the genome at a different location) [25], or by segmental or tandem duplication of a genomic region [26]. These are called processed or unprocessed pseudogenes, respectively. While processed pseudogenes in general have a single exon and a polyadenine tail, unprocessed pseudogenes typically have multiple exons and preserve the intron–exon structures of the parental gene. The vast majority of processed pseudogenes are “dead on arrival,” due to the lack of complete coding regions or necessary transcription and translation signals in the new genomic location. Even when a functional gene is formed by segmental duplication, one copy often becomes silenced by degenerative mutations due to functional redundancy [27]. In contrast, adaptive gene losses arise from degradation of genes with well-established functions, which often do not have close homologs in the genome. Taking advantage of the genomic signatures left behind by retrotransposition or gene duplication, several genomic surveys identified tens of thousands of pseudogenes in the human genome using sequence homology to a functional parental gene [28–32]. However, because they lack close homologs, many losses of well-established genes were missed by these studies. More importantly, these analyses focused on cataloging pseudogenes in the human genome, but not on addressing whether the pseudogenizations played a role in evolution.

This study identified losses of well-established protein-coding genes in the human lineage since the common ancestor of euarchontoglires (primates, lemurs, tree shrews, rodents, and lagomorphs such as rabbits). We applied a novel comparative genomic method to identify pseudogenes by syntenic mapping of gene structures between the human–mouse–dog trio of genomes. This approach is able to systematically detect the sequence signature left by losses of well-established genes, distinguishing true losses from mere loss of redundant genes following duplication or retrotransposition. Our analysis was able to differentiate the losses of well-established genes from the large background of human pseudogenes. Twenty six losses of well-established genes were identified in the human lineage since the common ancestor of euarchontoglires, approximately 75 million years ago (Mya). Sixteen of those were previously uncharacterized gene losses in the human genome, such as the loss of acyltransferase 3 during great ape evolution.

## Results

### Detecting Gene Losses Using Gene Structure Conservation

**Figure 1:**
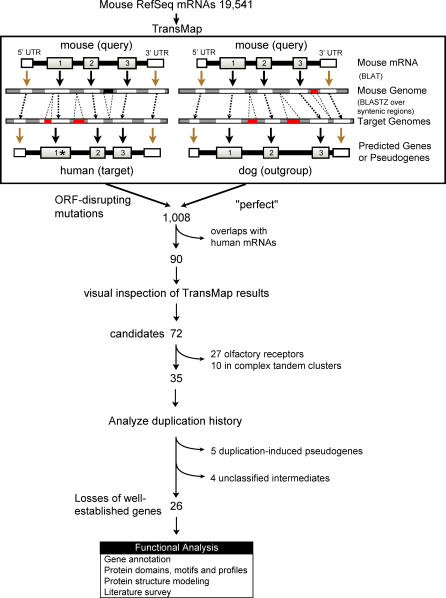
Algorithm for Identifying Losses of Well-Established Genes in the Human Lineage Since the Common Ancestor of Euarchontoglires TransMap predicts (pseudo)genes in the human and dog genomes by syntenically mapping mouse mRNA gene structures to the target genomes through genome alignments and then transferring over the corresponding genomic coordinates. The predicted coding regions are conceptually translated and scanned for ORF-disrupting mutations. In this example, a stop codon (labeled with an “*”) is detected in the first coding exon mapped to the human genome, which has also experienced an insertion. Genomic insertions or deletions are shown as red rectangles. Of 19,541 mouse RefSeq genes, 1,008 are identified as initial candidate gene losses in the human lineage based on the differential mutation status in the TransMap results. The list is narrowed down to 72 after eliminating those overlapping with human transcription evidence and filtered out by a manual inspection. Twenty-six are identified as losses of well-established genes in the human lineage after analyzing their duplication histories.

After a mutation inactivates a functional gene, the signature of the intron–exon structure can still be detected for some time before neutral decay erases it from the genome. Based on the observation that mammalian gene structures are typically conserved between species, a gene prediction program called TransMap was developed that exploits the large-scale conservation of gene order and orientation on mammalian chromosomes to map gene structures between genomes. TransMap is essentially a cross-species mRNA alignment grogram (Text S1) that relies upon the “syntenic” alignments produced by the BLASTZ program [33]. TransMap is highly sensitive in detecting gene structures for both genes and pseudogenes (Table S2). Unlike most existing pseudogene detection methods [30,31,34–36], TransMap does not rely on sequence homology to a parental gene from the same genome; therefore, it is well-suited for detecting losses of well-established genes whose functional precursor has not been recently duplicated. To identify gene losses, the mapped coding region is conceptually translated and scanned for ORF-disrupting mutations. A TransMap prediction is a candidate for gene loss if any of the following ORF-disrupting mutations are detected in the mapped coding regions: stop codons, frameshifts, a splice junction that is not GT-AG, or when less than 50% of the coding region can be mapped to the target (Figure S1). To decrease the number of false positives, a valid conceptual translation is required in an outgroup genome. For example, if a mouse mRNA TransMaps from mouse to the dog genome with a valid conceptual translation, but fails to do so from mouse to the human genome, the gene is a candidate for a human gene loss (Figure 1).

Using mouse mRNAs as queries and the dog genome as an outgroup, we identified gene losses in the human lineage since the common ancestor of euarchontoglires. We chose the RefSeq database because it is one of the most comprehensive collections of mouse transcripts [37]. Based on the differential prediction status in the dog and human genomes, 1,008 of the 19,541 mouse RefSeq transcripts were identified as potential human losses (Table S3). The candidates were reduced to 90 after filtering out overlaps with GenBank human mRNAs in order to identify pseudogenes where no transcriptional activity in the form of mature mRNA has been observed in humans, and to remove the false positive pseudogenes predicted by TransMap ((Text S1). A visual examination of an alignment of the mouse mRNA sequence, mouse, human, and dog genomic sequences and their three-frame translations further reduced the number of candidates to 72, eliminating 18 that we are not confident represent true pseudogenes in the human genome. The pipeline is illustrated in Figure 1.

###  Gene Losses in the Human Lineage

Of the 72 candidates, 27 are predicted to be olfactory receptors (ORs) and ten are members of large gene families, such as keratins. These gene families are organized as tandem gene clusters that have experienced copy number changes and/or complex local rearrangements since the common ancestor of euarchontoglires. The dynamics of gene clusters make it difficult to unambiguously discern ortholog/paralog relationships among species and analyze the evolutionary history of the lost genes. Therefore, we focused on the remaining 35 (non-OR, non-cluster) candidates that were confirmed by visual inspection, which we referred to as the “definite” losses in the human lineage. Among them, 21 gene losses are annotated with some biological functions in mouse and 14 have not been characterized functionally (Table 1).

**Table 1:**
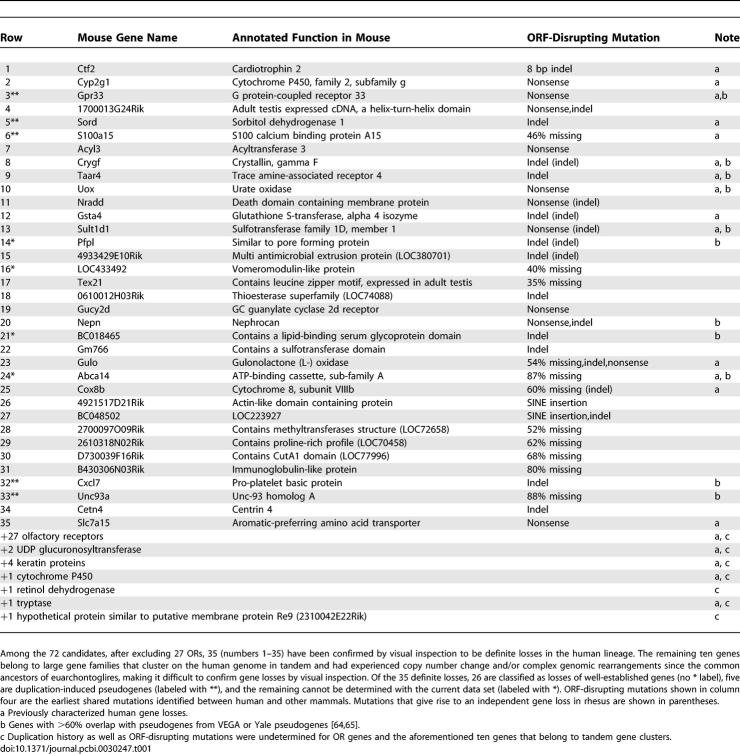
Gene Losses in the Human Lineage since the Common Ancestor of Euarchontoglires

The large number of ORs found by this method is consistent with observations that human OR genes experienced a rapid acceleration of pseudogene formation [38,39]. Previous studies have shown evidence that human genes involved in olfaction have a significant tendency to be under positive selection, indicating ORs have undergone directional selection in humans, including by pseudogenization [18,39]. Many previously identified non-olfactory gene losses were confirmed as well. These include gulonolactone (L-) oxidase (*GULO*), a vitamin C biosynthesis enzyme that is the genetic basis for scurvy [40], and urate oxidase (*UOX*), an enzyme converting uric acid to allantoin [41]. In addition, a human-specific nonsense mutation was confirmed in an orphan chemoattractant G protein–coupled receptor 33 (*GPR33*). Additionally, confirmed gene losses included the human-specific loss of cardiotrophin-2 (*CTF2*) due to a 8 bp deletion [42], cytochrome c oxidase subunit VIIIb (*COX8B*) with only 40% mapping to the human genome yielding an ORF of eight amino acids [43], and others listed in Table 1, note a.

**Figure 2:**
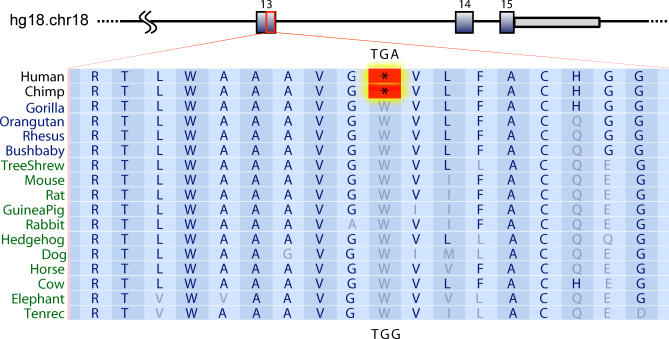
A Nonsense Mutation Inactivated *ACYL3* During Great Ape Evolution It occurred after the divergence of gorillas from the human lineage and before the human–chimp split. The nonsense mutation is located in exon 13 of *ACYL3*. A multispecies syntenic alignment showing the nonsense mutation (“*”) lies in a highly conserved protein coding region. The stop codon mutation (TGA) is present in the human and chimp genomes, but a TGG tryptophan (W) codon is present in the rhesus, mouse, rat, dog, and other mammalian genomes. The region maps to human Chromosome 18 at location 54,881,070–54,881,124. We sequenced the genomic region in a gorilla DNA sample to show that the codon TGG (W) codon is present in the gorilla genomes.

Our analysis also identified 23 previously uncharacterized losses, of which 21 belong to the definite loss group and two are members of complex gene clusters. One example of a previously unknown definite loss is acyltransferase 3 (*ACYL3*; NM_177028), identified by the Riken mouse cDNA project [37,44] and whose function in mammals has not been characterized experimentally. We conducted protein profile analysis and determined that this gene has a highly conserved acyltransferase 3 domain (hence we annotated the gene *ACYL3*) [45]. Further structural modeling (SAM, TMHMM, SignalP) revealed mouse Acyl3 is a multipass transmembrane protein with its C-terminal domain forming a helix bundle. The N-terminal is extracellular and hydrophilic with conserved cysteine residues able to form disulfide bonds [46–49]. The extracellular domain might be involved in cellular response to external signals. Thus, Acyl3 might be a membrane protein with acyltransferase activity or a multipass transporter to pass molecules across the membrane upon external signals [50,51]. Phylogenetically, *ACYL3* is ancient and conserved in archaea, bacteria, fungi, worms, flies, and mammals. While numerous copies of *ACYL3* are encoded in fly and worm genomes, mammalian genomes have only one copy. A nonsense mutation (TGG to TGA) located in one of the transmembrane helices is shared by the human and chimpanzee genomes. However, the ancestral TGG codon is present in two orangutan trace sequences, and a valid conceptual translation is present in the rhesus genome. To narrow down the timing of the inactivation, we sequenced a PCR product amplified from the corresponding region in a gorilla DNA sample. The sequencing result showed the TGG (W) codon is present in the gorilla genome. Based on this evidence, it appears that the nonsense mutation inactivated *ACYL3* after the divergence of gorillas from the human lineage, and before the divergence of humans and chimpanzees (Figure 2). It is intriguing that the last copy of such an ancient enzyme as *ACYL3* was lost during the evolution of great apes. Although we do not know the precise evolutionary impact of the loss, its expression pattern in the mouse pituitary gland and developmental abnormalities observed in *Drosophila* null mutants suggests the loss might be related to development or hormonal regulation [52–54].

As shown in Table 1, other previously unknown gene losses include *CETN4*, a mammalian centrin expressed in ciliated cells including those present in the cerebellum [55], *NEPN* (nephrocan), an inhibitor of TGF-β signaling pathway [56], and *NRADD*, a death domain containing membrane protein involved in mediating apoptosis in response to ER stress [57]. It is worth noting that ER-mediated apoptosis triggers a cascade leading to the activation of Caspase 12 (*CASP12*), a gene that is also lost in humans. The loss of *CASP12* is still polymorphic in humans and has been shown to have experienced a recent selective sweep [58,59].

### Timing of the Gene Losses

**Figure 3:**
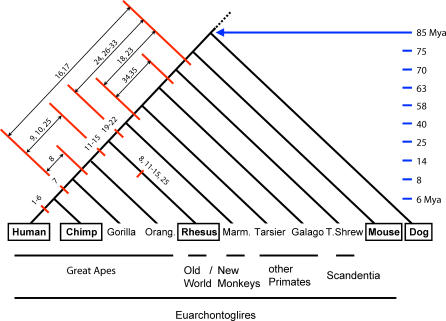
Timing of the Gene Losses in the Human Lineage Since the Common Ancestor of Euarchontoglires, Estimated Based on Shared Mutation Analysis Branch intervals enclosing the earliest ORF-disrupting mutations shared between human and other mammals are illustrated on the human lineage of a mammalian species tree. Genes are represented by numbers, which correspond to their row numbers in Tables 1 and 2. Marks on the rhesus lineage represent independent ORF-disrupting mutations that are not shared with the ones in the human lineage. Approximate time when the species diverged from the human lineage is shown in Mya. Species with complete genome sequences are enclosed by rectangles, while others only have trace sequences available for analysis. Orang.: orangutan; Marm.: marmoset; T.shrew: tree shrew.

The timing of the gene losses was determined by finding the branch interval that encloses the earliest shared ORF-disrupting mutations between humans and other mammals on a phylogenetic tree. The branch intervals on the human lineage for the 35 definite losses are illustrated on a mammalian phylogeny (Figure 3).

**Table 2:**
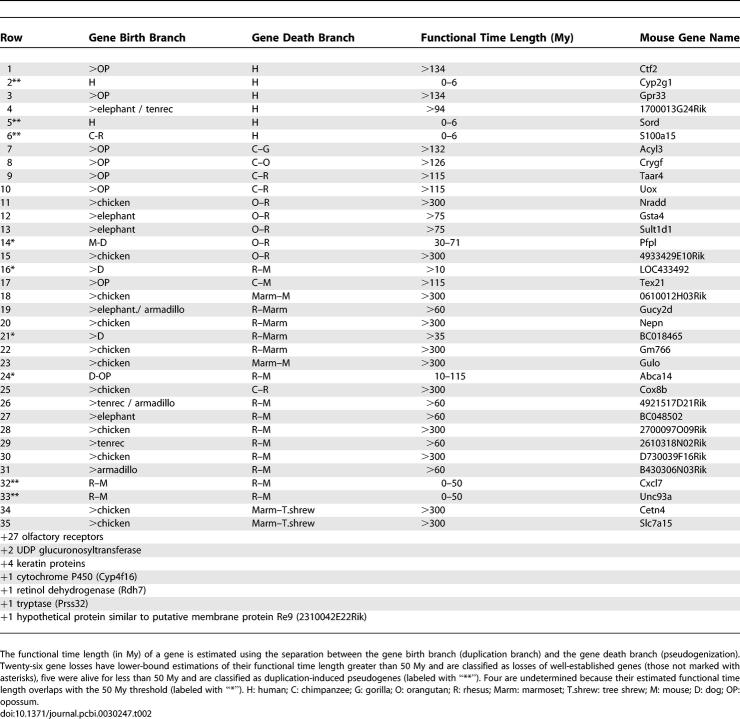
Losses of Well-Established Genes in the Human Genome, Classified Using Estimated Functional Time Length

Using complete genome sequences of human, chimp, rhesus, mouse, and dog, six genes were determined to be human-specific losses, i.e., lost after the divergence of humans and chimpanzees. Ten genes were found to be lost during the period between the human–chimp split and the divergence of old world monkeys from the human lineage. Among the ten genes, seven were observed to have independent ORF-disrupting mutations in the rhesus lineage that are not shared with humans or chimps. Seventeen genes were determined to be lost prior to the divergence of old world monkeys from the human lineage and after the common ancestor of euarchontoglires. Due to insufficient sequence information in the rhesus genome, two genes could only be determined to be lost at some point during the 70 million years (My) prior to the human–chimp split and after the common ancestor of euarchontoglires. To refine the timing of the gene losses, we extracted trace sequences from several additional primates (orangutan, marmoset, tarsier, galago) and tree shrew. Using these trace sequences, we were able to narrow down 50% of gene losses to a much more precise branch on a phylogenetic tree. For example, the timing of gene losses for *GUCY2D*, *NEPN*, and others (number 19 to 22) was narrowed down to the period of approximately 25 to 40 Mya, between the dates when old world and new world monkeys split off from the human lineage (Figure 3).

In addition to identifying previously unknown gene losses in the human genome, our analysis refined the timing of several previously known gene losses. For example, *SULT1D1*, a sulfotransferase, and *GSTA4*, a glutathione S-transferase alpha 4 isozyme are known to be pseudogenes in human while their mouse orthologs remain functional [60,61]; however, it was unclear when the inactivation occurred. Our analysis discovered that the pseudogenization of *GSTA4* and *SULT1D1* occurred approximately 14 to 25 Mya, between the dates when orangutans and old world monkeys split off from the human lineage. *GULO* is known to be inactive in primates with the inactivation dating to some time prior to the separation of apes and old world monkeys (>25 Mya) [62]. Frameshift indels, nonsense mutations, and genomic deletions are observed in the human *GULO* sequence, indicating an older pseudogene that has experienced numerous secondary mutations. The shared mutation analysis has shown that human, chimp, rhesus, and marmoset share a nonsense mutation, while galago, mouse, and dog share the TGG tryptophan codon. Therefore, the inactivation of *GULO* occurred before the separation of new world and old world monkeys (>40 Mya).

### Losses of Well-Established Genes versus Duplication-Induced Pseudogenes

A significant contribution of this analysis is to differentiate losses of well-established genes from the large background of pseudogenes caused by retrotransposition or formed shortly after segmental or tandem duplication. The method of syntenically mapping gene structures to both a target and outgroup genome is likely to filter out almost all processed pseudogenes. However, TransMap does not fully eliminate those genes that were silenced soon after duplication, which we referred to as duplication-induced pseudogenes. To identify duplication-induced gene losses, we need to determine when the duplication occurred.

**Figure 4:**
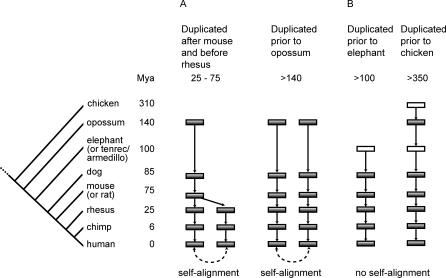
Timing of Gene Birth Is Estimated by Determining Duplication Histories of Genomic Regions Surrounding the Gene Losses For the subset of gene losses with detectable human self-alignment, the duplication branch is determined by tracing each duplicate of the best human self-alignment through a seven-species syntenic genomic alignment. For those without detectable self-alignments, the duplication branch is determined by the seven-species syntenic alignments plus alignments with the chicken genome, and the elephant, tenrec, and armadillo scaffolds when available. Filled rectangles represent syntenic alignment to the human genome, and open rectangles represent genomes or scaffolds aligned to the human genome without the syntenic constraint. Approximate time when the species diverged from the human lineage is shown in Mya.

Recent segmental duplications can be detected by within-genome sequence homology. If there is a self-alignment chain in the UCSC human genome browser [63] enclosing the gene loss region, the region is determined to have been recently duplicated. We determined when the duplications had occurred by tracing along the human lineage through a seven-species syntenic alignment (human, chimp, rhesus, mouse, rat, dog, opossum) to determine the origin of each duplicate in the best self-alignment (the one with the highest alignment score recorded in the UCSC genome browser). If a gene and its duplicate trace back to a single region in an outgroup genome, the duplication was determined to have occurred on the branch immediately after the outgroup split off from the human lineage. If the gene and its duplicate consistently traced back to different regions through the series of outgroups all the way back to opossum, the duplication was determined to occur prior to the common ancestor of human and opossum. Figure 4A is a schematic illustration of this procedure. The branch of gene duplication is the branch of gene birth (by duplication).

In many cases, there are no detectable self-alignments, indicating an ancient duplication had formed the functional precursor to the pseudogene. We presume the functional precursor of the pseudogene existed prior to the earliest common ancestor of human and the species whose genomic sequence can be aligned to the human exons, therefore providing a lower bound timing of the gene birth event. To narrow down the timing of gene birth on the long branch between dog and opossum, we included scaffold assemblies of the elephant, tenrec, and armadillo genomes. To infer gene birth events that occurred further back in the evolutionary past, we included the chicken genome in the analysis (Figure 4B). Using the above method, the gene birth by duplication branch for the 35 non-OR, non-cluster definite gene losses was determined and is shown in Table 2 (Gene Birth Branch).

Subsequently, we estimated the length of time (in My) a gene remained functional before its pseudogenization using the separation of the gene birth and death branches. Since the timings of both events are estimated as branch intervals, an upper and lower bound estimation of the separation was obtained. Using a 50 My threshold, we classified the candidates based on their functional time lengths as losses of well-established genes, duplication-induced pseudogenes, or undetermined. If the lower estimation of functional time length is greater than 50 My, the candidate is classified as a loss of a well-established gene. If the upper estimation of functional time length is smaller than 50 My, the gene loss is classified as a duplication-induced pseudogene. The gene loss is classified as undetermined if its functional time length overlaps the 50 My threshold.

Table 2 gives the estimated functional time length for the 35 definite losses. Among them, 26 are classified to be losses of well-established genes, which accounted for the majority (74%) of the definite losses. Five are classified as duplication-induced losses—*CYP2G1*, *SORD*, *S100a15*, *CXCL7*, and *UNC93a*— (labeled “**” in Tables 1 and 2). The remaining four are undetermined. Of these 26 losses of well-established genes, 16 have not been previously characterized as human pseudogenes in the literature. Among these 16, four have been functionally characterized in mouse, which are *NRADD*, *NEPN*, *CETN4*, and *GUCY2D*. Table S5 describes various subsets constructed using the 35 “definite” losses. All four candidates do not have detectable homologs in the human genome. Most strikingly, *NRADD*, *NEPN*, and *CETN4* remained functional for more than 300 My before being inactivated.

## Discussion

This study presents the first attempt to systematically identify adaptive gene losses in the human genome since the common ancestor of euarchontoglires, approximately 75 Mya. Using losses of well-established genes as the proxy for adaptive gene losses, we focused on identifying a class of pseudogenes that were once functional and retained this function through tens of millions of years of evolution. We confidently identified 26 losses of well-established genes, including 16 that were not previously known in the literature. The highlight of this analysis is the ability to automatically detect losses of genes bearing no significant homology to any functional paralog in the human genome. Their functional precursors had an ancient origin, but enough evolutionary time has elapsed to erase any significant homology with other genes in the human genome. These genes were functioning for hundreds of millions of years and silenced recently within the past 75 My.

It has been proposed that the majority of pseudogenes are either dead-on-arrival [58] or inactivated quickly after duplication [27]. Therefore, it is not surprising that we have identified a much smaller number of pseudogenes as compared to the thousands identified by previous whole genome analysis that aimed to catalog the human genome for unprocessed pseudogenes [30,31,36,64]. We overlapped our results with two well-known pseudogene databases, Yale pseudogene database, composed of mostly various computational predictions [64], and VEGA pseudogene collection, compiled by manual curation [65]. We found limited overlap between the losses identified in Table 1 with both pseudogene sets (Table S4). Only two out of 31 annotated, zero out of 14 hypothetical, and five out of 27 ORs were found by all three analyses. Neither database has *GULO*, Cardiotropin 2, or many others listed in Table 1 (see note b). A recent genome scan identified 67 human-specific gene losses, including 36 ORs [58]. Excluding ORs, only one out of the six human-specific gene losses identified in Table 1, Gpr33, was also discovered in that study [58]. Another possible overlap is Ugt2b1, which belongs to a tandem cluster of Ugt2B genes on Chromosome 4. The limited overlap in part reflects the difference in methodology used to identify the pseudogenes, but also makes apparent that none of these methods in their present state are able to form the complete set of losses of genes with ancient origins. It also confirms that we have identified some unprocessed pseudogenes derived from functional precursors of ancient origin, where evolution has erased any significant homology to their current functional paralogs.

The gene loss candidates shown in Table 1 are by no means a complete list of losses of well-established genes in the human lineage during the past 75 My. TransMap gene model prediction methodology is not perfect, many factors can introduce prediction errors including uncertainties in sequence alignments, errors generated by the gene model prediction and evaluation procedures, and evolutionary changes of the gene structures across mammalian species (Text S1). For example, the well-known human specific loss of *CMAH* (a CMP-sialic acid hydroxylase) [66] was not found by this analysis due to the strictness of TransMap gene model predictions, causing a valid *CMAH* gene model in the dog genome to be excluded because it featured a noncanonical GC-AG splice junction. However, the use of an outgroup genome and the mRNA filter makes the analysis far more likely to produce false negatives than false positives. Several other factors also contribute to this incompleteness. First, our method using human–mouse–dog comparison relied upon well-defined mouse genes to seed the search and valid dog predictions for outgroup confirmation. Problems in either one will return a false negative result. Our analysis missed *MYH16* because it is not in mouse RefSeq, which could be due to an independent loss or a misannotation. We further investigated its absence and found that the *MYH16* syntenic region is not present in the mouse genome, indicating an independent loss in mouse via genomic deletion. Our analysis required a valid conceptual translation in the dog genome, which may fail to occur due to TransMap prediction errors, sequencing gaps, or an independent loss in dog. However, the chance of producing a valid mapping increases if multiple outgroups, such as the opossum genome [67] or a computationally reconstructed ancestral genome [68], were used and the resultant gene loss predictions were combined. For example, the previously documented human specific loss of Htr5b [69] can be identified using a reconstructed boreoeutherian genome as the outgroup (Haussler lab, unpublished data). Our analysis can also be improved by extending our seed mRNAs to include those from other species and by using multiple outgroup genomes. For example, using chimpanzee *MYH16* mRNA as a seed could have found this pseudogene in human.

Our analysis may not identify human polymorphic gene losses. For example, the human-specific loss of *CASP12* [58,59] was not identified by our analysis because the latest human genome assembly (NCBI release 36) has the functional allele. Several other human polymorphic losses were also missed by our analysis for the same reason [70,71]. These polymorphic null alleles are potentially crucial to human diseases, e.g., *CASP12* in sepsis and *CCR5* in HIV infection. Incorporating human EST and mRNA information, as was done by Hahn et al. [70,71], or the human SNP dataset [72], could help our method identify human polymorphic gene losses. Overlapping those alleles with human disease loci, such as those documented in OMIM database [73] or identified by genetic association studies, might lead to the identification of new human disease associated genes. Another factor that may cause the method to overlook gene losses is related to segmental duplication. After a gene is duplicated, both the ancestral copy (the copy in the original genomic context) and the daughter copy (the copy duplicated in the new genomic context) are equally subject to degenerative mutations. Since our analysis evaluates based on the status of the ancestral copy, if evolution silences the daughter copy, it will not be identified by our method. However, this type of false negative is quite limited in our results because it only applies when a segmental duplication occurred after the boreoeutherian common ancestor. Treating the daughter copy in the same way as the ancestral copy will solve this problem, except in the case of a tandem segmental duplication, where it is difficult to distinguish the ancestral copy from the daughter copy.

Among the 26 losses of well-established genes, six were identified to be lost independently in the human and old world monkey lineages (numbers 8, 11, 12, 13, 15, 25 in Table 1). This can be interpreted as a confirmation for adaptive evolution, if we believe that a common selection pressure forced these genes to be lost in separate clades. Other known independent losses such as Caspase15 and Gpr33 seem to confirm this hypothesis [74,75]. An alternative interpretation is that the gene function is no longer needed, such as the loss of *GULO* in guinea pigs and humans [40]. However, it is also quite probable that the original loss did not occur independently on different lineages, but rather a common mutation that was missed by the analysis might have occurred earlier on a shared ancestor to inactivate the gene. This might have been a mutation in a noncoding region, or a mutation that was erased by secondary mutations such as genomic deletions. For example, a prior, noncoding mutation in any of the six cases we found could have disrupted the transcription, translation, or regulatory signals of the gene in the common ancestor of old world monkeys and apes, rendering the gene effectively inactive at the time that these lineages split. Since the gene is no longer under selective pressure to maintain its integrity, secondary ORF-disrupting mutations could follow, occurring independently in the separate lineages, as observed by our analysis.

To identify genes that are truly lost, we have focused on regions lacking any reported mRNA evidence, including in cell lines derived from cancer cells. A large number of candidates with differential mutational status in the human and dog gene predictions (918 out of 1,008) were filtered out because they overlap with some mRNA evidence in humans. The majority of these are likely to be TransMap prediction errors (Text S1, Table S1). However, some pseudogenes still generate transcripts if the transcription signal is intact, and these would be overlooked by our method. An example of a transcribed pseudogene in the human genome that appears on this list is *CATSPER2* (chr15: 41815434–41825788), represented by GenBank mRNA BC066967, and BC047442. The mammalian gene collection annotates it as a transcribed pseudogene. If a pseudogene is transcribed and spliced, its mRNA transcript with ORF-disrupting mutations (i.e., premature stop codon) is targeted and degraded by the cell's RNA surveillance pathway of nonsense mediated decay [76], although this process may not be complete. Only with time will these pseudogenes will be completely silenced at the level of transcription. In addition, studies have shown that occasionally a pseudogene, like Makorin1, not only transcribes but also plays a vital biological role in stabilizing the mRNA of its homologous coding gene [77]. Thus it is difficult to prove that a transcribed pseudogene is completely nonfunctional.

Theories of molecular evolution suggest three outcomes for new genes arising from gene duplication: degeneration due to functional redundancy, evolution into a new function, or function sharing by both copies [27]. The expected time that elapses before a gene is inactivated is thought to be relatively short [27]. Lynch and Conery estimated the half-life of a new duplicate to be around 16 My in the human lineage [27,78,79]. Using this estimate, after our cutoff of 50 My, 11% of redundant genes caused by duplications are expected to be intact by chance. After 60 My (the shortest estimation that passes the cutoff in Table 2), only 7.5% will be left. Twenty-six candidates in Table 2 are classified as losses of established genes using the 50 My cutoff, and many have an estimated functional period after duplication that is much longer than 50 My. This suggests that they are likely to have evolved independent functions before pseudogenization and thus likely to be true losses of well-established genes. In addition, our method used the lower-bound estimation for the functional time length for this classification. Although the higher-bound estimations for four candidates (*PFPL*, *ABCA14*, *LOC344492*, *BC018465* in Table 2) satisfy the 50 My cutoff, their low estimations do not. As complete genome sequences for additional mammals become available in the future, the timing of duplication and pseudogenization can be greatly refined, potentially classifying some of these four candidates as losses of established genes as well.

It is nontrivial to determine whether these losses we have found were truly adaptive. It is very likely that neutral losses at dispensable loci account for a subset of our results. For example, *GULO*, a vitamin C biosynthesis gene, is thought to have been lost in primates because primates have ample dietary supply of ascorbic acid, reducing or removing the selective pressure that maintains this gene. In general, it is difficult to differentiate between neutral loss due to removal of selective pressure, as proposed by the “use it or lose it hypothesis,” and positively selected adaptive loss, as by the “less is more” hypothesis, without knowing the gene's precise biological functions. Given our current knowledge of human genes, identifying the losses of established genes seem to be the best strategy in the search for more ancient (before 250 Mya) adaptive gene losses on a genomic scale. The resulting list is a much more enriched set of candidates.

In summary, our analysis identified a set of losses that are highly enriched for well-established genes in the human genome against a large background of pseudogenes. Expanding these results to include genes and genomes from the entire mammalian clade will generate a more accurate and comprehensive picture of adaptive gene losses in human evolution. From a theoretical standpoint, it will provide insight into the role that loss of functional genes plays in evolutionary adaptation [4]. The method presented here can also be generalized to discover gene losses in other organisms on a genomic scale.

## Materials and Methods

### Mapping mouse mRNAs to the human and dog genomes.

BLAT [80] alignments of mouse mRNAs sequences from Genbank [81] and RefSeq [37] to the cognate genome were obtained from the UCSC Genome Browser Database [82]. These alignments, along with the coding sequence annotations associated with the mRNAs, provide annotations of gene structure in the genome. BLASTZ [33] syntenic chained alignments [63] of cognate genome (mouse) to the target genome (human or dog) are used to project the mouse mRNA alignments to the target genome. This algorithm, known as TransMap and illustrated in Figure 1, results in predictions of orthologous gene structures in the target organism. The TransMap prediction methodology is described in detail in Text S1.

### Sources of sequences, mRNAs, and pseudogene databases.

Genomic sequences used in this study were obtained from the UCSC genome browser [82]. Sequence release of the following species are human (NCBI release 36.1; UCSC hg18 March 2006), chimp (Chimpanzee Sequencing and Analysis Consortium Build 2 version 1; UCSC March 2006), rhesus macaque (BCM HGSC version 1.0, Mmul_051212; UCSC Jan 2006), mouse (NCBI release 36; UCSC Feb 2006), dog (Broad Institute assembly version 2.0; UCSC May 2005), rat (Baylor Human Genome Sequencing Center HGSC version 3.4; UCSC Nov 2004), opossum (draft assembly produced by the Broad Institute; UCSC Jan 2006), chicken (version 2.1 draft assembly produced by the Genome Sequencing Center at the Washington University; UCSC May 2006), elephant (Broad Institute version 1.0; UCSC May 2005), tenrec (Broad Institute echTel 1.0; UCSC Jul 2005), and armadillo (Broad Institute version 1.0; UCSC May 2005). Trace sequences of orangutan, marmoset, tarsier, galago, and tree shrew were downloaded from NCBI Trace Archive. Mouse RefSeq genes were obtained from the UCSC mouse genome browser, which is consistent with the NCBI mouse genome build 36.1. Yale and VEGA pseudogene datasets were also obtained from the UCSC human genome browser track “Yale Pseudo” and track “Vega Pseudogenes.” Human mRNAs filter is the GenBank human mRNAs, obtained from the UCSC human genome browser track “Human mRNAs”. The identifiers of the two orangutan trace sequences used to confirm the TGG (W) codon is present in the orangutan genome are ti865941905 and ti1012155976 in the NCBI trace archive.

### *ACYL3* domain analysis and structural modeling.

The mouse gene *ACYL3* (NM_177028) is predicted to contain an acyltransferase 3 (acyl3, IPR002656) and a nose resistant to fluoxetine-4 (NRF, IPR006621) domain in the InterPro database [45]. We predicted *ACYL3* to have eight or nine transmembrane helixes in its C-terminal sequence using TMHMM v2.0 [48] and to have a 20 amino acid signal peptide in its N-terminal sequence by SignalP 3.0 [49]. We performed protein structure modeling using SAM-T05 [46]. SAM (dssp-eh12 model) predicted *ACYL3* to be a multi-pass membrane protein with four conserved cysteine residues in its N-terminal sequence and 12 helixes in its C-terminal sequence. The nonsense stop codon shared by humans and chimpanzees is located in the tenth helix of the structural prediction.

### DNA amplification and sequencing of ACYL3 region in gorilla.

Genomic DNA surrounding the *ACYL3* TGG (W) codon was PCR-amplified from a gorilla DNA sample. Degenerate PCR primers were designed based on the conservation in human, chimp, and rhesus genomic sequences. The forward primer is 5′-GGTCACCCTATTTGCGGTGGCCGCTCTGGCATACA-3′ and the reverse primer is 5′-TGGGCTGGGTCCTCTTTGCGTGCCACNGAGGATATGGAGGTATGGA-3′. The PCR product was sequenced in both forward and reverse directions, and results were combined to generate a 162 bp gorilla sequence.

### Determining earliest shared mutations.

The earliest shared mutations were determined by examining an alignment of genomic sequences of human, chimp, rhesus, mouse, and dog, plus 200 bp trace sequences surrounding the mutation site, when available in the NCBI trace archive, from orangutan, marmoset, tarsier, galago, and tree shrew. Trace sequence analysis was limited to candidates with only point mutations (i.e., stop codons, frameshift indels, or noncanonical splice sites). Candidates with coverage problems (>50% missing) were only analyzed to check whether the limited coverage is also shared by the chimp or rhesus genomes, and their point mutations were not analyzed except for a stop codon mutation in *GULO*.

## Supporting Information

Figure S1 A Histogram of the Number of TransMap Gene Models Predicted in the Human Genome against the Fraction of the Length of the Mouse mRNA Coding Sequence That Can Be Aligned (21 KB PDF)

Table S1 Evaluation of the TransMap Gene Model Classifications against ENCODE Gene Annotations (52 KB DOC)

Table S2 Comparison of Sequence Coverage by TransMap and Other Sequence Aligners (22 KB DOC)

Table S3 The Percentage of Alignments or Gene Models with a “Valid” Code Assignment (20 KB DOC)

Table S4 Genomic Coordinates of the Lost Genes and Their Overlap with YALE and VEGA Pseudogenes (58 KB DOC)

Table S5 Description of the Various Subsets Constructed Using the 35 “Definite Losses” (27 KB DOC)

Text S1 Description of the TransMap Gene Prediction Methodology (49 KB DOC)

## Abbreviations

My: million years
Mya: million years ago
OR: olfactory receptor
