# Supplementary material for: Comparative Genomics Search for Losses of Long-Established Genes on the Human Lineage
Source: PLoS Comput Biol. 2007 Dec 14;3(12):e247. doi: 10.1371/journal.pcbi.0030247 (PMC2134963; doi:10.1371/journal.pcbi.0030247)
Supplement: Supplementary file 1 [file pcbi.0030247.sg001.pdf]

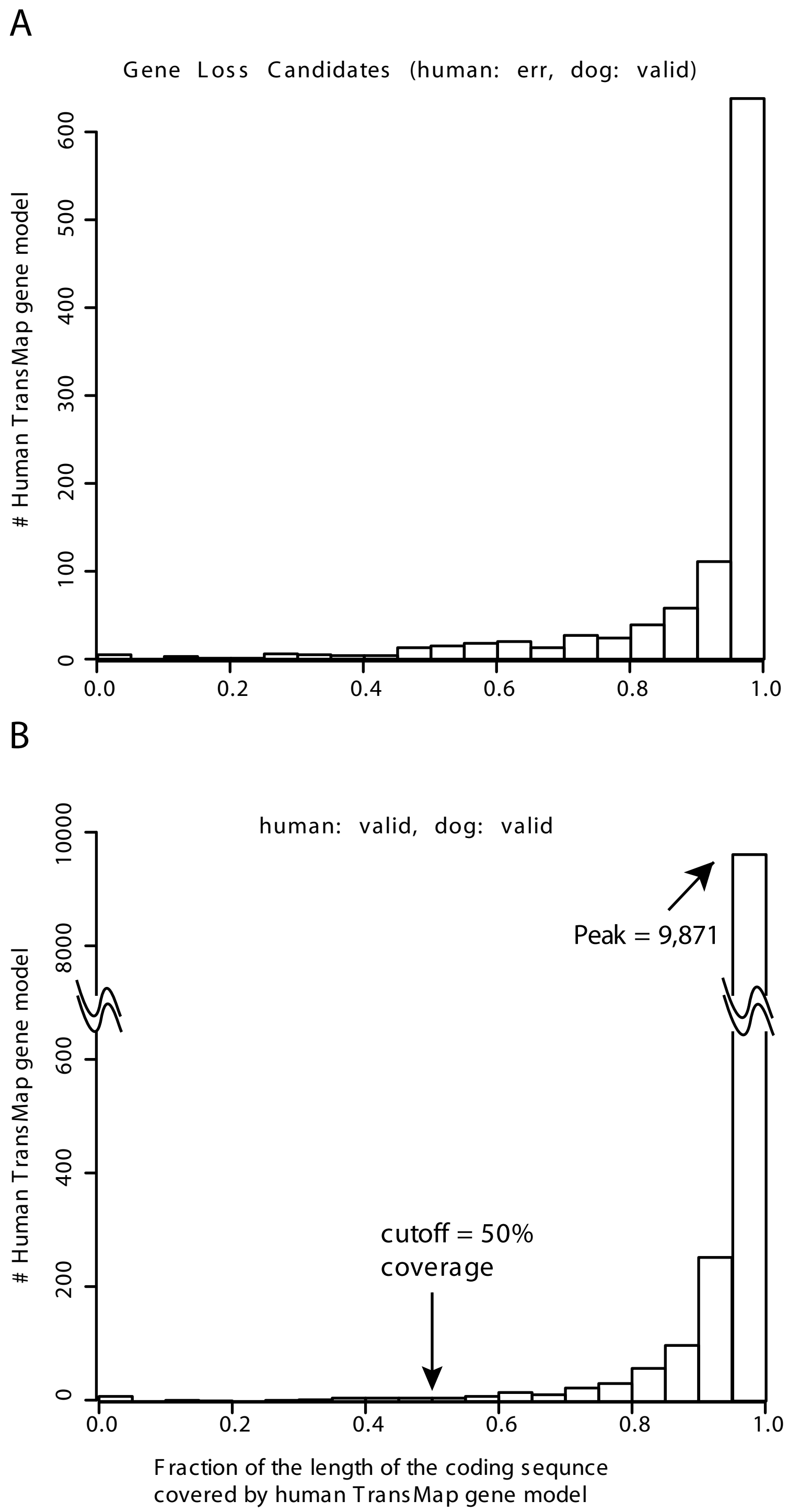
A
Gene Loss Candidates (human: err, dog: valid)
Human TransMap gene model
0
100
200
300
400
500
600
0.0
0.2
0.4
0.6
0.8
1.0
B
human: valid, dog: valid
Human TransMap gene model
0
200
400
600
8000
10000
Peak = 9,871
cutoff = 50% coverage
0.0
0.2
0.4
0.6
0.8
1.0
Fraction of the length of the coding sequnce covered by human TransMap gene model
