# Supplementary material for: Comparative Genomics Search for Losses of Long-Established Genes on the Human Lineage
Source: PLoS Comput Biol. 2007 Dec 14;3(12):e247. doi: 10.1371/journal.pcbi.0030247 (PMC2134963; doi:10.1371/journal.pcbi.0030247)
Supplement: Supplementary file 2 [file pcbi.0030247.sd001.doc]

**Text S1 TransMap gene prediction methodology**

Taking advantage of the conservation of gene structures across mammalian genomes, the TransMap algorithm predicts gene models in a target genome that are orthologous to the source genes in the cognate genome. The key operation of this algorithm is syntenic mappings of gene structures across genomic sequences to produce such a gene model.

The TransMap methodology consists of three steps. The mapping step aligns an mRNA sequence to the cognate genome and then projects this alignment to the target genome, resulting in a cross-species mRNA alignment. The gene prediction step adjusts these alignments to compensate for evolutionary changes in order to produce a gene model. The evaluation step determines whether the gene model is valid, assigning a code of “valid” or “err” accordingly. The final TransMap prediction is a gene model plus the associated evaluation code.

**Alignment mapping step**

The mapping step creates an alignment of an mRNA from its source species to the genome of a different target species. The first step of the mapping aligns an mRNA sequence to its cognate genome using the BLAT program [1]. BLAT is designed to align transcripts of at least 95% identity to DNA sequences, producing intron-spanning alignments of the full cDNA. The second step of the mapping projects these mRNA alignments to the genome of a target species via BLASTZ [2].  BLASTZ is a highly sensitive cross-species aligner optimized for aligning diverged, orthologous genomic sequences. The BLASTZ alignment nets are used to select alignment chains that are classified as syntenic [3] to remove paralogous alignments. This projection results in a cross-species mRNA alignment in the target genome, similar to what one does with protein translated BLAT [1] or TBLASTN [4]. See table S2 for a comparison of TransMap with these methods.

For this analysis, BLAT alignments of mouse RefSeq [5] mRNAs to the mouse genome (NCBI build 36, UCSC mm8) were obtained from the UCSC Genome Browser database [6]. These alignments, along with the coding region annotations associated with the mRNAs, provide annotations of gene structure in the mouse genome. BLASTZ chained alignments of mouse source genome to the human (NCBI build 36, UCSC hg18) and dog (Broad 2.0, UCSC canFam2) target genomes are used to project the mouse mRNA alignments to the target genomes. The BLASTZ alignments are also obtained from the UCSC Genome Browser database. We select the subset of the UCSC alignment nets (mouse to human, mouse to dog) that are classified as syntenic by the netFilter program. These are either first-level nets of length longer than 20Kb or nets of subsequent levels of length greater than 30Kb.

**Gene model prediction step**

The TransMap algorithm is highly sensitive at detecting the approximate gene structures in the target genome (table S2). However, a TransMap alignment (i.e. cross-species mRNA alignment projections) may not be perfect or realistic as a gene model for various reasons, including evolutionary change of the gene structures across species, errors introduced via BLAT and/or BLASTZ alignments, or errors in the original mRNA sequences. A set of heuristic adjustments is applied to produce a gene model from a TransMap alignment. For example, when the alignment projection does not have a start codon at the beginning of the projected coding region, the heuristic routine searches the neighborhood of the start site to find an in-frame ATG codon that does not introduce a premature stop codon. A similar operation is carried out to find the GT-AG splice junction. Exon sequence divergence can often lead to alignment gaps, so the heuristics attempt to close such gaps to produce a continuous exon sequence. In the end, these heuristic adjustments attempt to produce a gene model, although these adjustments are not perfect and some errors are still likely to be present.

**Gene model evaluation**

The predicted gene model is subsequently evaluated to determine whether or not it is a valid gene model. The following conditions are evaluated: a start codon at the beginning of the coding sequence, a stop codon at the end of the coding sequence, dinucleotides GT and AG respectively located at the splice donor and acceptor sites, and a continuous coding sequence lacking premature stop codons or frameshifts. If all criteria are satisfied, the gene model produces a perfect conceptual translation and is assigned a “valid” evaluation code; otherwise, an “err” code is assigned.

In this analysis, we used the evaluation code as the proxy for gene and pseudogene classifications. The accuracy of TransMap assignment is evaluated using the ENCODE gene and pseudogene classifications. The results are described in table S1. In summary, when TransMap assigns a “valid” code, the chance of the assignment being correct is 98%. However, when it assigns an “err” code, the chance the assignment being correct is 24%. 1,008 candidates were compiled as the initial human pseudogene set in this analysis using the TransMap evaluation code. The majority of them are not really pseudogenes in the human genome, due to the low negative prediction value. An example is the gene model that maps to human *SNX26.* This error results from evolutionary change in the gene structure. The human sequence corresponding to the mouse coding sequence start site has been changed from ATG to ACG. Human *SNX26* uses an alternative start codon located in a different exon in the 5’direction; however, TransMap heuristic adjustment is unable to find the alternative start codon. This results in the gene model being assigned an “err” code. We subsequently used the human GenBank mRNA set to remove the vast majority of these false positives. Although the negative predictive value (the fraction of true pseudogenes of all models that are assigned an “err” code) is in itself far from perfect, the human mRNA filter and visual inspection assures the downstream candidates are truly pseudogenes in the human genome. These filters, combined with the requirement of a valid dog gene model, make the strategy far more likely to produce false negatives than false positives.

Because the large majority (98.7%, see reference Nucleic Acids Res. 2001 Jan 1;29(1):255-9. SpliceDB: database of canonical and non-canonical mammalian splice sites. [7]) of the donor and acceptor splice sites follows the canonical dinucleotide sequence, our current implementation of TransMap only considers the GT-AG junction as the valid sequence. This simplification will miss real splice junctions that feature noncanonical junctions, such as the case for the mapping of mouse *CMAH* to the dog genome, because the splice junction of the first and second coding exons is a non-canonical GC-AG sequence (in all three genomes: mouse, dog and human). As a result, this human-specific gene loss ended up as a false negative for our procedure.

Altogether, we speculate the following factors contribute to errors in TransMap code assignment (“valid” vs. “err”):

- Errors in mouse RefSeq mRNA sequences
- BLAT alignment error introduced in the process that aligns mouse mRNAs to the mouse genome
- BLASTZ alignment error introduced in the process that aligns mouse genome to the human or dog genomes
- Errors in selecting syntenic alignment chains
- Limitations of TransMap’s assumption that gene structures are canonical and conserved through mammalian evolution, while in fact a gene structure occasionally does change across mammalian species.

These leave room for future improvements to the method.

## Reference

1. Kent WJ (2002) BLAT--the BLAST-like alignment tool. Genome Res 12: 656-664.

2. Schwartz S, Kent WJ, Smit A, Zhang Z, Baertsch R, et al. (2003) Human-mouse alignments with BLASTZ. Genome Res 13: 103-107.

3. Kent WJ, Baertsch R, Hinrichs A, Miller W, Haussler D (2003) Evolution's cauldron: duplication, deletion, and rearrangement in the mouse and human genomes. Proc Natl Acad Sci U S A 100: 11484-11489.

4. Altschul SF, Gish W, Miller W, Myers EW, Lipman DJ (1990) Basic local alignment search tool. J Mol Biol 215: 403-410.

5. Pruitt KD, Tatusova T, Maglott DR (2007) NCBI reference sequences (RefSeq): a curated non-redundant sequence database of genomes, transcripts and proteins. Nucleic Acids Res 35: D61-65.

6. Kuhn RM, Karolchik D, Zweig AS, Trumbower H, Thomas DJ, et al. (2007) The UCSC genome browser database: update 2007. Nucleic Acids Res 35: D668-673.

7. Burset M, Seledtsov IA, Solovyev VV (2001) SpliceDB: database of canonical and non-canonical mammalian splice sites. Nucleic Acids Res 29: 255-259.
