# Supplementary material for: Comparative Genomics Search for Losses of Long-Established Genes on the Human Lineage
Source: PLoS Comput Biol. 2007 Dec 14;3(12):e247. doi: 10.1371/journal.pcbi.0030247 (PMC2134963; doi:10.1371/journal.pcbi.0030247)
Supplement: Supplementary file 3 [file pcbi.0030247.st001.doc]

**Table S1** Evaluation of the TransMap gene model classifications against ENCODE gene annotations

|  | | ENCODE annotation | |  |  |
| --- | --- | --- | --- | --- | --- |
|  |  | Gene transcripts | Unprocessed pseudogenes | N | Predictive values |
| TransMap gene model evaluation code | Valid | 284 | 5 | 289 | 98.3% = 284/ 289  (positive predictive value) |
|  | Err | 74 | 24 | 98 | 24.5% = 24/98  (negative predictive value) |

The TransMap code assignments are evaluated using ENCODE region gene and pseudogene classifications. The ENCODE genes are the published Gencode transcripts for the human genome ENCODE region [1,2]. The ENCODE unprocessed pseudogenes are those annotated as the consensus of five pseudogene prediction algorithms [3]. The TransMap human gene models and their associated assignment codes are produced by TransMapping mouse mRNAs to the human genome. A TransMap gene model is evaluated to be “valid” when it can produce a satisfactory conceptual translation using the TransMap evaluation routine. Otherwise, the gene model is assigned an ”err” code. The analysis is carried out on the subset of genes or pseudogenes that intersects the TransMap gene models and ENCODE annotations.

1. Harrow J, Denoeud F, Frankish A, Reymond A, Chen CK, et al. (2006) GENCODE: producing a reference annotation for ENCODE. Genome Biol 7 Suppl 1: S4 1-9.

2. Birney E, Stamatoyannopoulos JA, Dutta A, Guigo R, Gingeras TR, et al. (2007) Identification and analysis of functional elements in 1% of the human genome by the ENCODE pilot project. Nature 447: 799-816.

3. Zheng D, Frankish A, Baertsch R, Kapranov P, Reymond A, et al. (2007) Pseudogenes in the ENCODE regions: consensus annotation, analysis of transcription, and evolution. Genome Res 17: 839-851.
