# Supplementary material for: Comparative Genomics Search for Losses of Long-Established Genes on the Human Lineage
Source: PLoS Comput Biol. 2007 Dec 14;3(12):e247. doi: 10.1371/journal.pcbi.0030247 (PMC2134963; doi:10.1371/journal.pcbi.0030247)
Supplement: Supplementary file 4 [file pcbi.0030247.st002.doc]

**Table S2** Comparison of sequence coverage by TransMap and other sequence aligners

|  | Mouse | Human | | |
| --- | --- | --- | --- | --- |
|  | BLAT | XBLAT | TBLASTN | TransMap |
| Number of transcripts aligned | 20456 | 20031 | 20013 | 18558 |
| Rate of transcripts aligned | 1.000 | 0.979 | 0.978 | 0.907 |
| Rate of multiple alignments | 0.006 | 0.071 | n/a | 0.040 |
| Fraction of 5’ UTR aligned | 0.994 | 0.325 | n/a | 0.841 |
| Fraction of coding sequence aligned | 0.998 | 0.869 | 0.365 | 0.923 |
| Fraction of 3’ UTR aligned | 0.997 | 0.236 | n/a | 0.777 |

The table compares the results of aligning of 20,456 mouse RefSeq mRNAs to the human genome. The BLAT column is the filtered BLAT alignments to the mouse genome, obtained from the UCSC Genome Browser database. XBLAT is protein-translated BLAT to human. TBLASTN is the alignments of the protein coded for by the mRNAs to human using the translated BLAST to nucleotide algorithm. TBLASTX is not used due to memory requirements. The cross-species alignments are filtered to keep only those alignments scoring within the top 1% of the best alignments for a given mRNA. Due to TBLASTN producing independent exon alignments, local near-best filtering is used and the rate of multiple alignments is not computed.
