# Supplementary material for: Comparative Genomics Search for Losses of Long-Established Genes on the Human Lineage
Source: PLoS Comput Biol. 2007 Dec 14;3(12):e247. doi: 10.1371/journal.pcbi.0030247 (PMC2134963; doi:10.1371/journal.pcbi.0030247)
Supplement: Supplementary file 5 [file pcbi.0030247.st003.doc]

**Table S3** The percentage of alignments or gene models with a “valid” code assignment

| Source gene | Mouse mRNA | Mouse mRNA | Mouse mRNA |
| --- | --- | --- | --- |
| Target genome | Mouse genome | Human genome | Dog genome |
| Mapping method | Raw BLAT alignment | Gene model by TransMap | Gene model by TransMap |
| % with a “valid” code assignment | 85% | 77% | 68% |

The code assignments are generated using the TransMap gene model evaluation routine.
