# Supplementary material for: Comparative Genomics Search for Losses of Long-Established Genes on the Human Lineage
Source: PLoS Comput Biol. 2007 Dec 14;3(12):e247. doi: 10.1371/journal.pcbi.0030247 (PMC2134963; doi:10.1371/journal.pcbi.0030247)
Supplement: Supplementary file 6 [file pcbi.0030247.st004.doc]

**Table S4** Genomic coordinates of the lost genes and their overlap with YALE and VEGA pseudogenes

| **Pseudogene ID** | **HG18 Location** | **Vega/Yale Pseudogene Overlap (> 60%)** | **Mouse Annotation (if any)** |
| --- | --- | --- | --- |
| NM_001008499.1-1.1 | chr6:132957223-132958268 | V/Y | Taar4 trace amine-associated receptor 4 |
| NM_007751.2-1.1 | chr11 243417 243558 | Y | Cox8b cytochrome 8, subunit VIIb |
| NM_008159.1-1.1 | chr14:31021882-31026992 | V/Y | Gpr33 G protein-coupled receptor 33 |
| NM_009474.3-1.1 | chr1:84603188-84636156 | V | Uox urate oxidase |
| NM_010357.1-1.1 | chr6:52912485-52945892 | V | Gsta4 glutathione S-transferase, alpha 4 |
| NM_010663.1-1.1 | chr17:20356269-20361411 | Y | Krt1-17 keratin complex 1, acidic, gene 17 |
| NM_013707.1-1.1 | chr21:30730324-30731174 | Y | Krtap14 keratin associated protein 14 |
| NM_013809.1-1.1 | chr19:46247785-46260189 |  | Cyp2g1 cytochrome P450, family 2, subfamily g |
| NM_015741.1-1.1 | chr17:36622484-36623047 | Y | Krtap9-1 keratin associated protein 9-1 |
| NM_016771.2-1.1 | chr4:70687437-70720060 |  | Sult1d1 sulfotransferase family 1D, member 1 |
| NM_017473.1-1.1 | chr12:55661222-55666095 | Y | Rdh7 retinol dehydrogenase 7 |
| NM_019540.1-1.1 | chr11:58810633-58814011 | Y | Pfpl pore forming protein-like |
| NM_019784.3-1.1 | chr14:63879902-63915769 |  | Tex21 testis expressed gene 21 |
| NM_023785.1-1.1 | chr4:74932359-74933982 |  | Cxcl7 pro-platelet basic protein |
| NM_024442.1-1.1 | chr19:15535443-15556847 | Y | Cyp4f16 cytochrome P450, family 4, subfamily f |
| NM_026012.2-1.1 | chr3:47026634-47029966 |  | Nradd death domain containing membrane protein NRADD |
| NM_027010.1-1.1 | chr2:209718174-209720719 | Y | Crygf crystallin, gamma F |
| NM_027220.1-1.1 | chr16:2789020-2795066 |  | Prss32 tryptase 5 |
| NM_028094.1-1.1 | chr4:70142918-70151231 | Y | Ugt2a3 UDP glucuronosyltransferase 2 family, |
| NM_145825.1-1.1 | chr4:123870718-123873126 |  | Cetn4 centrin 4 |
| NM_146126.1-1.1 | chr15:42905239-42941396 |  | Sord sorbitol dehydrogenase 1 |
| NM_152811.1-1.1 | chr4:69904790-69920682 | Y | Ugt2b1 UDP glucuronosyltransferase 2 family |
| NM_177802.2-1.1 | chr2:20455521-20460982 |  | Slc7a15 aromatic-preferring amino acid transporter |
| NM_178747.2-1.1 | chr8:27473714-27503386 |  | Gulo gulonolactone (L-) oxidase |
| NM_198858.1-1.1 | chr16:30822866-30829066 |  | Ctf2 cardiotrophin 2 |
| NM_199422.1-1.1 | chr1:151635327-151638220 |  | S100a15 S100 calcium binding protein A15 |
| NM_177028.2-1.1 | chr18:54814918-54887549 |  | Acyl3 acyltransferase 3 |
| NM_199252.1-1.1 | chr6:167541894-167555675 |  | Unc93a unc-93 homolog A |
| NM_001034890.1-1.1 | chr11:76074935-76084205 |  | Gucy2d (LOC434214 hypothetical protein LOC434214) |
| NM_025684.1-1.1 | chr6:118061568-118080190 | V | Nepn (5730521E12Rik hypothetical protein LOC66650) |
| NM_026458.3-1.1 | chr16:21857687-21863975 | Y | Abca14 ATP-binding cassette, sub-family A |
| NM_001025574.1-1.1 | chr20:31397095-31406542 |  | LOC433492 hypothetical protein LOC433492 |
| NM_001033417.1-1.1 | chr12:22135947-22179281 |  | Gm766 hypothetical protein LOC330440 |
| NM_001033542.1-1.1 | chr17:19423766-19477673 |  | 4933429E10Rik hypothetical protein LOC380701 |
| NM_025634.1-1.1 | chr3:185699692-185701043 |  | 2310042E22Rik hypothetical protein LOC66561 |
| NM_026338.1-1.1 | chr3:49885419-49889756 |  | 4921517D21Rik hypothetical protein LOC67722 |
| NM_027063.1-1.1 | chr1:22149751-22152304 |  | 1700013G24Rik hypothetical protein LOC69380 |
| NM_028314.1-1.1 | chr14:34493816-34521518 |  | 2700097O09Rik hypothetical protein LOC72658 |
| NM_028747.1-1.1 | chr11:32089736-32311979 |  | 0610012H03Rik hypothetical protein LOC74088 |
| NM_030021.1-1.1 | chr9:122,649,393-122,655,217 |  | D730039F16Rik contain CutA1 domain |
| NM_144890.1-1.1 | chr20:31368330-31384061 | V | BC018465 hypothetical protein LOC228802 |
| NM_177083.2-1.1 | chr6:41257342-41262067 |  | B430306N03Rik hypothetical protein LOC320148 |
| NM_177631.2-1.1 | chr12:53163432-53172287 |  | BC048502 hypothetical protein LOC223927 |
| NM_177717.2-1.1 | chr12:51088354-51101918 |  | 4732456N10Rik similar to KERATIN, TYPE II CYTOSKELETAL 6D |
| NM_183287.2-1.1 | chr22:20336505-20345277 |  | 2610318N02Rik hypothetical protein LOC70458 |
| NM_001011759.1-1.1 | chr11:56036282-56037222 | Y | Olfr1031 olfactory receptor 1031 |
| NM_001011784.1-1.1 | chr11:55936796-55937753 | Y | Olfr1039 olfactory receptor 1039 |
| NM_001011785.1-1.1 | chr11:56569084-56569425 | Y | Olfr987 olfactory receptor 987 |
| NM_001011801.1-1.1 | chr10:45073628-45074622 | V/Y | Olfr213 olfactory receptor 213 |
| NM_001011844.1-1.1 | chr1:156978558-156979829 | V/Y | Olfr426 olfactory receptor 426 |
| NM_010990.1-1.1 | chr11:49901388-49902000 | Y | Olfr48 olfactory receptor 48 |
| NM_010991.1-1.1 | chr14:22241192-22242128 | V/Y | Olfr49 olfactory receptor 49 |
| NM_019473.1-1.1 | chr9:36003162-36012259 | V/Y | Olfr155 olfactory receptor 155 |
| NM_146322.1-1.3 | chr3:99423590-99424512 | Y | Olfr187 olfactory receptor 187 |
| NM_146354.1-1.1 | chr11:5155568-5156419 |  | Olfr633 olfactory receptor 633 |
| NM_146356.1-1.1 | chr11:74519550-74521126 | Y | Olfr521 olfactory receptor 521 |
| NM_146363.1-1.1 | chr14:19385366-19386346 | V/Y | Olfr731 olfactory receptor 731 |
| NM_146485.1-1.2 | chr3:99423578-99424512 | Y | Olfr183 olfactory receptor 183 |
| NM_146490.1-1.1 | chr2:240668121-240668455 | Y | Olfr1411 olfactory receptor 1411 |
| NM_146568.1-1.1 | chr11:56264231-56265159 | Y | Olfr1012 olfactory receptor 1012 |
| NM_146585.1-1.1 | chr11:55936796-55937753 | Y | Olfr1517 olfactory receptor 1517 |
| NM_146647.1-1.1 | chr11:55379644-55380576 |  | Olfr1154 olfactory receptor 1154 |
| NM_146679.1-1.1 | chr11:59055803-59056743 | Y | Olfr1427 olfactory receptor 1427 |
| NM_146803.1-1.1 | chr11:123740721-123741657 | Y | Olfr906 olfactory receptor 906 |
| NM_146803.1-1.2 | chr11:123732656-123733605 | Y | Olfr906 olfactory receptor 906 |
| NM_146803.1-1.3 | chr11:123713861-123714804 | Y | Olfr906 olfactory receptor 906 |
| NM_147028.1-1.1 | chr11:55472225-55482476 | Y | Olfr1125 olfactory receptor 1125 |
| NM_147036.1-1.1 | chr12:47122050-47122997 | Y | Olfr283 olfactory receptor 283 |
| NM_147037.1-1.1 | chr2:240697185-240698160 | Y | Olfr1413 olfactory receptor 1413 |
| NM_147052.1-1.1 | chr11:5070482-5071433 | Y | Olfr589 olfactory receptor 589 |
| NM_147063.1-1.1 | chr11:74519556-74521126 | Y | Olfr520 olfactory receptor 520 |
| NM_206896.1-1.1 | chr2:240697185-240698186 | Y | Olfr12 olfactory receptor 12 |

**V**: overlap with VEGA pseudogenes. **Y**: overlap with Yale pseudogenes. A subset of the Yale pseudogenes is classified as unitary pseudogenes, i.e. pseudogenes resulting from the decay of a previously functional gene, which are similar to what we defined as losses of well-established genes. Overlapping with the Yale pseudogenes finds 12 (Taar4, Cox8b, Gpr33, Rdh7, Pfpl, Crygf, Cyp4f16, Ugt2b1, Ugt2a3, Krtap9-1, Krtap14, and Krt1-17) out of 31 annotated genes, zero out of 16 hypothetical genes, and 25 out of 27 olfactory receptors. At the time of the analysis, VEGA annotations covered chromosome 6, 7, 9, 10, 13, 14, 20, 22, and X. Overlapping with the VEGA pseudogenes finds 5 annotated (Uox, Gsta4, Taar4, Nepn and Gpr33), 1 hypothetical, and 5 olfactory receptor genes.
