# Supplementary material for: Comparative Genomics Search for Losses of Long-Established Genes on the Human Lineage
Source: PLoS Comput Biol. 2007 Dec 14;3(12):e247. doi: 10.1371/journal.pcbi.0030247 (PMC2134963; doi:10.1371/journal.pcbi.0030247)
Supplement: Supplementary file 7 [file pcbi.0030247.st005.doc]

**Table S5** Description of the various subsets constructed using the 35 “definite losses”

|  | | | **35** “definitive” losses | | | |  |  | | |
| --- | --- | --- | --- | --- | --- | --- | --- | --- | --- | --- |
|  |  |  | Losses of long-established genes  (> 50MY) | | Duplication-induced pseudogenes  (< 50MY) | Undetermined |  |  |  |  |
|  |  |  | 26 | | 5 | 4 |  |  |  |  |
| 35 “definitive”  losses | Previously known in the literature | 14 | 10 | | 3 | 1 |  | 21 | Functionally characterized in mouse | 35 “definitive”  losses |
|  | Unknown | 21 | 16 | (4) | 2 | 3 |  | 14 | Uncharacterized in mouse |  |
|  |  |  |  | (12) |  |  |  |  |  |  |

By determining gene duplication histories, we are able to differentiate losses of well-established genes from losses of recently duplicated genes. Using this method, we identified 26 losses of well-established genes (functional for at least 50 million years before loss), of which 16 have not been known previously in the literature. Within the 16, four have been functionally characterized in mouse.
